# Towards Interpretable Camera and LiDAR Data Fusion for Autonomous Ground Vehicles Localisation

**DOI:** 10.3390/s22208021

**Published:** 2022-10-20

**Authors:** Haileleol Tibebu, Varuna De-Silva, Corentin Artaud, Rafael Pina, Xiyu Shi

**Affiliations:** Institute of Digital Technologies, Loughborough University London, 3 Lesney Avenue, London E20 3BS, UK

**Keywords:** glass detection, occupancy grid mapping, LiDAR noise reduction, localisation

## Abstract

Recent deep learning frameworks draw strong research interest in application of ego-motion estimation as they demonstrate a superior result compared to geometric approaches. However, due to the lack of multimodal datasets, most of these studies primarily focused on single-sensor-based estimation. To overcome this challenge, we collect a unique multimodal dataset named LboroAV2 using multiple sensors, including camera, light detecting and ranging (LiDAR), ultrasound, e-compass and rotary encoder. We also propose an end-to-end deep learning architecture for fusion of RGB images and LiDAR laser scan data for odometry application. The proposed method contains a convolutional encoder, a compressed representation and a recurrent neural network. Besides feature extraction and outlier rejection, the convolutional encoder produces a compressed representation, which is used to visualise the network’s learning process and to pass useful sequential information. The recurrent neural network uses this compressed sequential data to learn the relationship between consecutive time steps. We use the Loughborough autonomous vehicle (LboroAV2) and the Karlsruhe Institute of Technology and Toyota Institute (KITTI) Visual Odometry (VO) datasets to experiment and evaluate our results. In addition to visualising the network’s learning process, our approach provides superior results compared to other similar methods. The code for the proposed architecture is released in GitHub and accessible publicly.

## 1. Introduction

Localisation is one of the critical aspects for autonomous ground vehicles (AGV) to operate safely. However, a reliable GPS signal is not always available in many scenarios, which makes localisation and orientation estimation of AVGs difficult, even impossible sometimes. A fully autonomous system requires dependable navigation capability that works in all environments, including where a strong GPS signal is available and in a condition in which a GPS signal is unreliable. Therefore, a mobile platform’s self-contained pose and orientation estimation are critical as they are fundamental steps to obtain vital navigation information for a mobile robot and autonomous vehicles. Autonomous systems collect essential information about their environment using onboard sensors, including cameras, LiDAR, inertial measuring unit (IMU), radar and digital compasses. This information is necessary for the autonomous system to track past trajectories, acquire current location and plan future movements. Odometry uses data from motion sensors to estimate the change in relative position of a mobile robot over time.

An efficient camera and LiDAR feature extraction method is essential for odometry estimation. In computer vision, a sequence of image and LiDAR frames can be used to extract features from structural motion. The captured environments move when LiDAR and camera sensors are in motion, resulting in a gradual intensity change between adjacent pixels. Effective extraction of the change in motion is the backbone of odometry estimation. Odometry can be estimated with a single or multiple sensors.

Camera and LiDAR sensors are by far the two most common methods chosen by the robotics community to tackle an odometry problem. Both sensors have their advantages and disadvantages. LiDAR has superb capability to obtain different features of the environment. LiDAR is also not affected by different lighting conditions. Most of all, LiDAR obtains more accurate range measurements than a camera. However, reflection heavily influences LiDAR data, and, unless using additional algorithms, LiDAR cannot detect glass [1]. Besides, LiDAR data are very sparsely distributed and have a limited visibility range. LiDAR also collects too much data, which requires high computational power for processing. It is also more expensive than a camera. 

Cameras provide dense and rich data, making them ideal for obstacle avoidance problems in AGVs. A camera is not affected by the presence of glass to the same degree as LiDAR and also does not have a limited visibility range. However, unless it is a 360° camera, it will have a narrow visibility angle, resulting in blind spots. Apart from RGB-D cameras, standard cameras do not measure distance. Cameras also struggle to work in adverse weather conditions and are also severely impacted by different lighting conditions. Other odometry methods are based on fusion of data from multiple sensors [2,3,4,5,6]. Feature-based algorithms use gematrically steady features and track them across consecutive frames [6,7]. The motion of the sensor is then tracked by estimating the minimised reprojection error across pairs of frames. Although this method is robust, it mostly fails in an environment that does not generate proper-fit features to be extracted. The other method is called a direct method [8], which traced the intensity of pixels across frames and estimated the camera’s motion from photometric error.

Current research has directed efforts towards deep-learning-based approaches. This method outperforms conventional approaches in related domains, such as object detection and classification [9,10]. It also yields better performance in odometry applications. However, it needs to be investigated further as it is still a new research area.

Research in Refs. [11,12] stated that the fusion method performs better odometry than a single-sensor approach. Both papers use CNN and LSTM network architecture. While Ref. [11] addresses the problem as a classification problem, Ref. [12] deals with this problem as a regression problem. Both methods yield better performance than conventional approaches. In this research, we collected a unique multimodal dataset—LboroAV2—from multi-sensors, including a camera, LiDAR, electronic compass, rotary encoder and ultrasonic sensors, suitable for research related to localisation and mapping. The data in LboroAV2 are acquired using our AGV while driving in multiple paths and sequences with ground truth data. We proposed efficient data-driven neural network architecture combining LiDAR and camera for odometer applications. The proposed architecture is tested using the KITTI dataset and LboroAV2.

The rest of the paper is organised as follows. A review of the literature is presented in the related work section. The methodology section describes details about the research methods and the developed localisation algorithms, followed by the results and discussion section. Last, we summarise our findings in the conclusion section. 

## 2. Related Work

Ego-motion, also known as pose estimation, is one of the primary challenges in simultaneous localisation and mapping (SLAM). Pose estimation plays a significant role in various applications, such as autonomous cars [2], unmanned air vehicles (UAV) [3], robotic hands [4] and 3D reconstruction [5]. Pose estimation is the key to tracking position over time. There are two approaches to odometer: traditional methods (geometry model) and learning methods. The traditional methods have shown superb performance, especially in reducing noise. However, they lack adaptability to all challenging scenarios. Moreover, the conventional methods depend on hand-craft modules. To overcome the shortcomings of the traditional approach, researchers have been proposing learning methods with promising results [6,7,8,9].

Most previous research based on learning methods used data from either range measurement or visual sensors. Only a few studies have used fusion of LiDAR and camera for odometry application.

### 2.1. Visual Odometry

There are two different approaches to achieve this: feature-based and direct methods [10]. These approaches target estimating the pose of a robot based on images. Feature-based methods follow the standard pipeline, including feature extraction, matching, estimation of motion and local and/or global optimisations [11,12]. Assuming that scenes are stationary, the direct method aims to minimise photometric error by considering all the pixels in the image [13,14]. The assumption in a direct method that scenes are stationary is not conclusive enough to be satisfactory. Hence, most direct methods have lower performance than feature-based methods. 

Following advancement in deep learning, researchers are exploring deep-learning-based approaches for odometry application using visual data, also known as visual odometry (VO) [6,7,8,9,15,16]. This technique requires ground-truth-labelled data. Some researchers use a pretrained FlownetS model [17] by remodelling it for prediction of relative poses [18,19]. The FlownetS model using a single architecture, given two consecutive RGB images, executes feature extraction and matching for pose estimation. This technique assumes that features learned from the two successive images for optical flow estimation can also be used to predict the relative pose between the images. 

Another approach uses optical flow as input and reverts the pose based on learned information from the past [6,19,20]. Deep learning approaches are not only learning visual signs but are also learning camera intrinsic features.

Wang et al. [21] argued that, while CNNs are suitable for capturing useful learning features, they are not satisfiable enough to extract motion dynamics. Hence, Wang suggested that CNN models are not good enough for visual odometry (VO). Therefore, Wang proposed sequential-based modelling with deep recurrent convolutional neural networks (RCNN). While the FlownetS architecture is augmented with LSTM, which outputs pose estimation for each time step, LSTM is not suitable for high-dimensional data. Ref. [22] proposed a stacked autoencoder to capture optical flow and predict relative poses of the camera by forming a multi-object loss function to minimise inaccuracy. This method performs better than traditional monocular approaches, similar to that of Ref. [12]. Nevertheless, the multi-objective may not share favourable features for both reconstruction and pose estimation. 

### 2.2. LiDAR Odometry

LiDAR odometry is the process of estimating the relative pose of two 3D point cloud frames [10]. The LiDAR apparatus is known for its accurate depth information. Nevertheless, using LiDAR data for odometry is challenging due to the sparse nature of point cloud data. Most LiDAR-based odometry works are based on traditional pipelines and achieved promising performance [23,24]. Martin et al. [25] proposed a method to overcome the shortcoming of LiDAR point cloud sparsity by grouping the LiDAR points into a polar bin and generating a line segment for each bin. Although it produces a better result than the traditional methods, it is computationally expensive and cannot be applied in real-time.

The deep learning method has also been used for the problem of LiDAR-based odometry. However, this method is still very challenging. For instance, Refs. [26,27] used CNN to perform LiDAR-based odometry. In this method, the original data are transformed into a dense matrix with three channels. While Ref. [26] only estimated translational data, Ref. [27] encoded the point cloud into data matrices to feed into the network, making direct data processing more practical. Ref. [28] used a deep neural network by replacing each component of the traditional pipeline. Other methods, such as DeepLO [29], LO-Net [27] and DeepVCP [30], design different types of end-to-end deep-learning-based frameworks and have been conducted using the supervised learning method.

### 2.3. Fusion of Visual and LiDAR Odometry

Research conducted by fusing visual and LiDAR data takes advantage of both the LiDAR and visual information to predict the translation and rotation of a mobile robot. Again, most of these methods are conducted using a traditional approach. V-LOAM [31] combines visual odometry and LiDAR odometry for better model optimisation using a conventional feature-based pipeline. LIMO [32] proposes feature-based visual odometry, which takes scale estimation from LiDAR data to attain pose estimation. 

Recent emerging research has used learning techniques to fuse LiDAR and visual data for odometry purposes. Ref. [33] used high-resolution images to improve 3D point clouds using the deep learning method. Ref. [34] proposed self-supervised visual odometry for estimation of scale-aware poses. However, they use only images as input to the network. The LiDAR data are added as a supplement. Ref. [10] proposed self-supervised LiDAR and visual odometry that takes monocular images and depth maps obtained from the LiDAR point clouds as network input to estimate pose. 

This research proposes deep learning architecture for real-time odometer applications, which can also be used to explain the learned features and process at the compressed representation mode. To the best of our knowledge, this is the first architecture that fuses LiDAR and camera data in the compressed representation node of the mono-modal encoders. We propose a new network architecture that provides a better result compared to similar approaches. The network architecture can be used to tackle other similar problems. We also collected a unique dataset named LboroAV2. The LboroAV2 data can be used for different localisation problems. The data are acquired in both structured and non-structured outdoor environments. We use the LboroAV2 and KITTI datasets for training and testing our model.

## 3. Method

This section first describes the LboroAV2 and KITTI VO datasets, followed by a detailed explanation of the proposed approaches. The proposed method combines the convolutional encoder with LSTM model to estimate translation and rotation between the fusion of two consecutive LiDAR scan and camera images. 

### 3.1. Dataset

A review of existing datasets that are potentially suitable for this research has been conducted. Except for a few, the majority of exiting datasets are mono-modal. Amongst a very few who have a multimodal dataset, none of the datasets have both structured and unstructured environments. Hence, this research investigates two datasets: the publicly available KITTI [35] and LboroAV2. The KITTI dataset is a famously known dataset for SLAM, visual and inertia odometry research. The main purpose of using LboroAV2 is to add environmental conditions that are undiscovered by KITTI dataset. The shortcomings of KITTI dataset are described in the discussion section of this paper. The KITTI dataset contains 20 sequences of LiDAR and a camera; however, the ground truth data are only available for 10 sequences.

The LboroAV2 multimodal dataset is collected specifically for this project. An AGV [36] is used to collect the dataset. The AGV was autonomously driven throughout the data collection period, with minimal human interaction, on structured and unstructured roads on the privately owned Here East compound in Queen Elizabeth Olympic Park, London. Multiple sensors are embedded in the AVG platform, including a camera, LiDAR, ultrasound, rotary encoder and electronic compass. 

The VLP-16 is a low-powered compact optical sensor with a useable range of up to 100 m. It utilises 16 pairs of emitter detectors, which measure a total of 300,000 data points per scan. 

**Figure 1:**
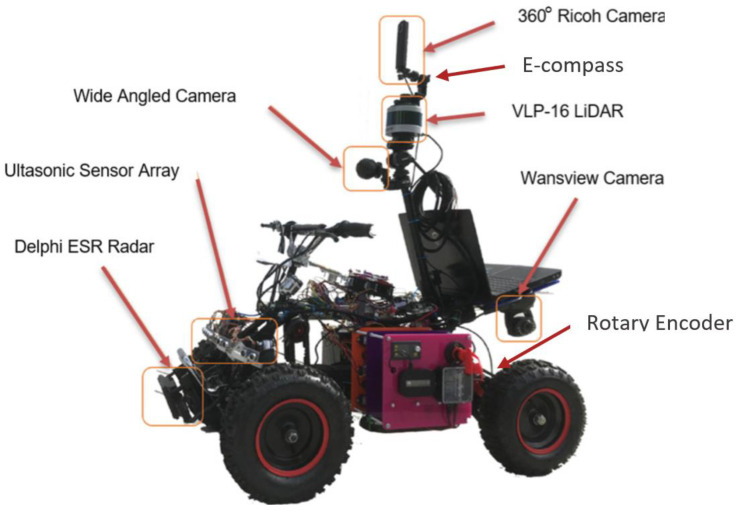
The unmanned ground vehicle used for LboroAV2 data collection [36].

The data were logged using two methods: directly from the sensors to a laptop and using data logger mounted on the AVG. There were 864 K (at 60 fps) frames captured by the wide-angle camera and 432 K frames (at 30 fps) captured by the Ricoh Theta V 360° camera. The rotary encoder and ultrasound sensor captured 62 K (5 fps) scans, respectively. The rotary encoder and electronics compass captured 144 K (10 fps) datasets each. Given that each sensor has its own limitations, the mixture offered by multimodality has excellent potential to sum up a positive contribution to the intended capability of any machine. Figure 1 shows the AGV used for data collection and the onboard sensor’s locations.

**Figure 2:**
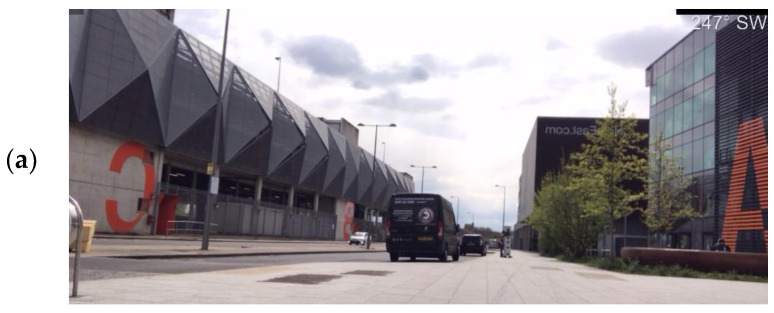

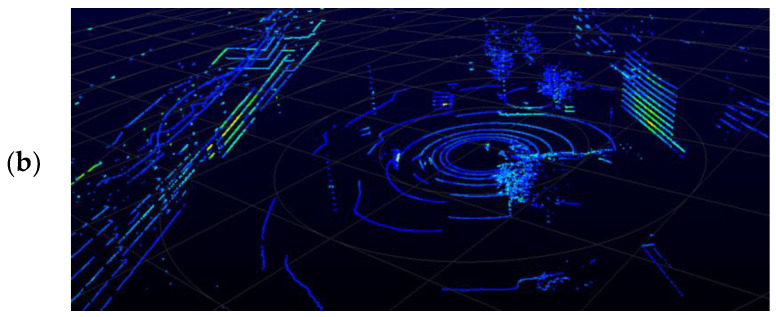
Sample of camera (**a**) and LiDAR (**b**) data from LboroAV2 dataset.

The LboroAV2 dataset was collected in four separate routes. The total distance of data collection covers over 1.2 km. The dataset contains multiple outdoor environments at different times of the day. The data collection period covers 11 months to include a wide range of class features, including pedestrians, cyclists and vehicle traffic in many different environmental conditions. Sample image and LiDAR scan from LboroAv2 dataset are presented in Figure 2.

The dataset considered in this research is selected based on the assortment of foreground and background objects and the overall scene layout. The sensor data not selected for this research are compiled for future work. 

Although the AVG platform’s operation speed can reach up to 22 km/h, its speed during the data collection period is limited to 4 km/h due to the license and permit approved by Here East management.

Sensor parts that are subject to a build-up of contaminants, such as lenses, were sufficiently maintained throughout the data collection period. A unique label for each sequence was added to the captured data—labels for accessible grouped data collected in the same sequence. Care was taken to ensure that all data captured were meticulously archived, labelled and stored securely following data protection acts.

### 3.2. Data Encoding

The data coming from LiDAR and camera sensors are required to be pre-processed before feeding to the proposed network architecture. The original image data from the KITTI and LboroAV2 datasets are transformed into a size of 416 × 128 pixels. The primary aim of compressing the datasets is to reduce the computational time of training while not losing valuable features. The LiDAR data are encoded in two methods, and each method is tested in the experiment. In the first method, we encode the LiDAR dataset based on [37]. The raw LiDAR data are initially binned with a resolution of 0.10. The 3D LiDAR data are then encoded into a 1D vector. We use the average value of point clouds that belong to the same bins as the encoded value, as defined in (1). The final vector has 3601 elements, which stores each bin’s depth information.

(1) $${Ꝕ}_{i}=\frac{1}{N}\sum_{k=0}^{N}\alpha_{k}\;,\;\forall i\in \left[0,\;3600\right]$$

where ${Ꝕ}_{i}$ represents the encoded value of *i*th bin, *α* is raw depth information and *N* is the total number of LiDAR data points in a single scan. 

Once each image and LiDAR data are encoded as described, each sequential LiDAR and camera data scan is concatenated. The images are concatenated channel-wise to give a sequence of 6 × 128 × 416. The LiDAR data become 2 × 3601 size after concatenation of two consecutive scans. The concatenated LiDAR shape enables the network to pass a convolutional layer for feature extraction. 

**Figure 3:**
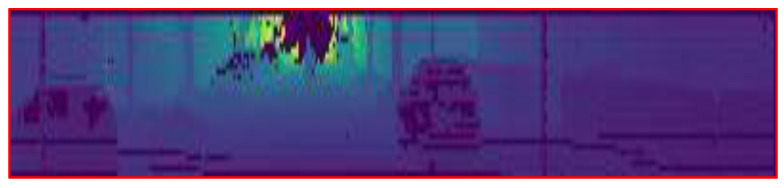
LiDAR point clouds projected onto a panoramic image.

The second LiDAR data encoding method is based on the approaches in Ref. [38]. The 3D LiDAR data are projected to a 2D image, as shown in Figure 3. The projected 2D image contains the local geometry of the 3D voxel space. This feature helps the model to learn the translation and rotation while reducing the input size of the model. We limit the projection of the LiDAR to the front side 180° field of view. 

### 3.3. Deep Learning Architecture

**Figure 4:**
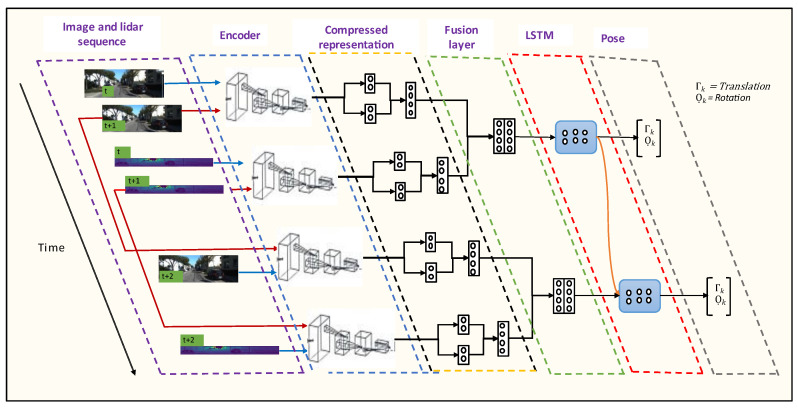
Architecture of the proposed end-to-end deep learning method.

Deep-learning-based algorithms have become common practice for image classification due to the availability of large-scale datasets. Applications such as odometry require a high volume of data suitable for learning geometric features representing the surrounding environments. Unlike traditional methods, deep learning approaches do not require manual data calibration. Although current deep learning approaches outperform traditional methods, their architectural designs are not good enough to grasp all the useful underlining features of the input data. Moreover, it is impossible to explain the underline feature extraction process between neurons as the machine learning process is thought as a black box problem. To overcome this limitation, we propose a new design of an end-to-end deep learning architecture, as shown in Figure 4, that can better extract features representing image and LiDAR data for odometer applications. The architecture contains two parts: an encoder that includes compressed representation node and a sequential modelling with RNN. The compressed representation node space of this model can also be used to gain insight into the deep learning network.

Our motivation is to propose not only a network that can estimate rotation and translation accurately but also a model that can be used to gain insight into the architecture’s learning process. Our method is also distinct in its ability to visualise fusion of represented features from camera and LiDAR sensors. This architecture helps to advance development of responsible AI.

**Figure 5:**
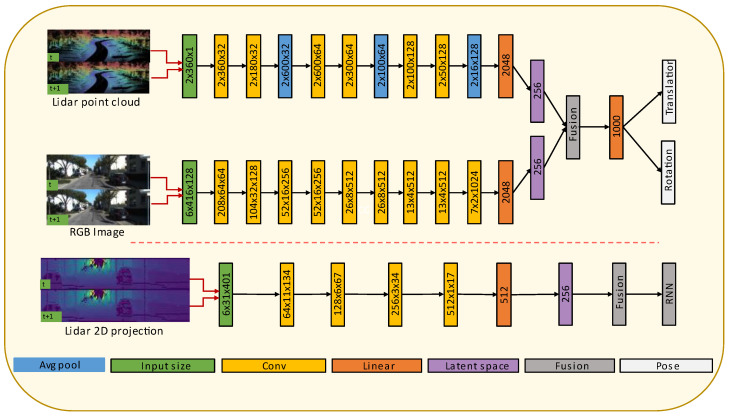
Architectures for fusion of camera and LiDAR point cloud data (upper part), and fusion of camera and LiDAR data projected in 2D images (lower part).

**Figure 6:**
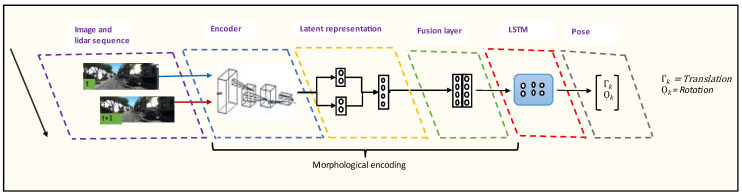
Proposed mono camera odometry architecture.

Three different architectures have been experimented with in the research. Figure 5 shows the architectures for fusion of camera and LiDAR point cloud data and fusion of camera and LiDAR data projected in 2D images. Figure 6 shows the camera only architecture. The architectural details of each model are discussed below.

#### 3.3.1. Encoder

Popular deep learning architectures, such as Alexnet [39] and VGG [40], are designed to perform well for tasks such as object detection and classification, primarily dependent on objects’ appearance in the image. However, training data for odometry applications require deeper extraction of geometric features from consecutive scans. We use different feature extraction architectures for the camera and LiDAR, which work based on the same principle, referred to as encoder hereafter. The detail of the encoder for each modality is described below. 

#### 3.3.2. Camera Feature Extraction

We modify the Flownets [17] optical flow estimation method, which uses two consecutive RGB images to extract useful features that help estimate the pixel flow between two images. The architecture is composed of 9 convolutional layers. Except for the last layer, all the convolutional layers are followed by rectified linear unit (ReLU) activation function. We gradually decrease the kernel size from 7 × 7 to 3 × 3. We use a stride of 2 for Conv 1–3, Conv 5, Conv 7 and Conv 9; for the rest, we use a stride of 1. We also apply padding that is decreased gradually from 3 to 1. At the end of the 9th convolution, the extracted features are further reduced to a vector of 256 elements, known as compressed representation node, as shown in Figure 6. This compressed representation node is a systematically condensed representation of the features in the original input data. 

#### 3.3.3. Point Cloud Feature Extraction

As described in the section of data encoding process (Section 3.2), the concatenated two LiDAR point cloud data scans have a shape of 2 × 3061. These data are then sequentially fed to a 1D convolution layer. We use six 1D convolutional layers following the method in Ref. [41]. Each convolutional layer is followed by a ReLU activation. Average polling is applied after each convolutional layer to further reduce the computational time. At the end of the last convolutional layer, the extracted features are further reduced to a vector of 256 elements, as shown in Figure 5, so that both the camera and LiDAR feature extraction have the same compressed representation node shape. 

#### 3.3.4. LiDAR Projected 2D Image Feature Extraction

The projected LiDAR 2D image size is smaller than the RGB camera image. Hence, we reduced the LiDAR feature extractor architecture. The projected 2D image has a shape of 3 × 31 × 401. The final size of the channel-wise concatenated 2D projected LiDAR image is 6 × 31 × 401. The proposed LiDAR projected 2D image feature extraction architecture is presented in Figure 5.

#### 3.3.5. LiDAR and Camera Fusion 

Once the LiDAR and camera data have the same size compressed representation node through their feature extractions, the fusion is conducted by concatenating each modality’s compressed representation nodes. The rotation and the translation have the same sized compressed representation node. The learned features in the compressed representation node have an effectively encoded representation and can enhance the efficacy of the sequential training method. 

#### 3.3.6. RNN Sequential Modelling

The first RNN-based visual odometry was proposed in Ref. [21]. RNNs are a branch of deep networks suitable for understanding the core features in a sequential dataset or a sensor data stream. The LboroAV2 data from multiple modalities contain a useful sequential relationship between consecutive scans. Thus, using RNN model for odometer application is necessary to exploit those temporal relationships between successive scans. However, it is not pertinent to learn temporal relationships using the raw image and LiDAR point cloud data as these data are very high-dimensional. Thus, in our architecture, we use the concatenated compressed representation of the LiDAR and camera data as input to the RNN. 

The unique feature of RNN is that it retains the memory of features output of the previous cell (hidden state) over time. Given feature $x_{t}$ at time t, the RNN updated at time t 

(2) $$h_{t}=\sigma \left(W_{xh\;}x_{t}+W_{hh}h_{t-1}+b_{h}\right)$$

(3) $$y_{t}=W_{hy\;}h_{t}+b_{y}$$

where $h_{t}\;$ is the hidden state and $y_{t}$ is output at time t. σ is an element-wise non-linear activation function. $W_{xh\;}$ is denoted by the weight matrices of the hidden layer. $b_{h}\;\mathrm{and}\;b_{y}$ represent the bias. 

Given a hidden state $h_{t-1\;}$ and the memory cell of the previous state $\;c_{t-1}$ at $x_{t}$ time t, the network updates as follows

(4) $$f_{t}=\sigma_{g}\left(W_{f}x_{t}+J_{f}h_{t-1}+b_{f}\right)$$

(5) $$o_{t}=\sigma_{g}\left(W_{o}x_{t}+J_{o}h_{t-1}+b_{o}\right)$$

(6) $$i_{t}=\sigma_{g}\left(W_{i}x_{t}+J_{i}h_{t-1}+b_{i}\right)$$

(7) $${Ć}_{t}=tanh\left(W_{Ć}x_{t}+J_{Ć}h_{t-1}+b_{Ć}\right)$$

where $f_{t}$, $i_{t}$, $o_{t}$ and ${Ć}_{t}$ represent input gate, output gate, forget gate and input cell states. $W_{f},\;W_{o},\;W_{i}\;\mathrm{and}\;W_{Ć}\;$ are the weight matrices of the three gates and the input cell states. $j_{f},\;j_{o},\;j_{i}\;\mathrm{and}\;j_{Ć}$ are the weight matrices of the input gate, output gate, forget gate and input cell states, whereas b and $\sigma_{g}$ are the bias vector and the gate activation functions.

The hidden state $h_{t}$ and the cell output states $C_{t}\;$ compute as follows

(8) $$h_{t}=o_{t\;}*\;tanh(C_{t})$$

(9) $$C_{t}=f_{t\;}*\;C_{t-1\;}+i_{t}*\left({Ć}_{t}\right)$$

**Figure 7:**
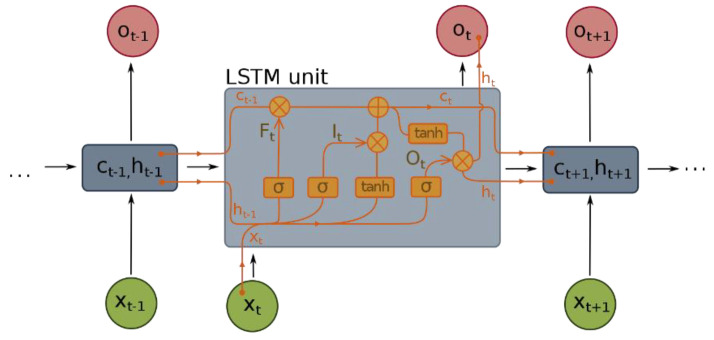
The hidden state interactions in an LSTM model.

Although the classic RNN can learn temporal information, it suffers from a vanishing gradient when the gradient passes over a long timestep. Long-short-term memory (LSTM) has been introduced to overcome this problem [42]. There are three gate systems in LSTM: an input gate, an output gate and a forget gate, represented as sigmoids (*σ*) or hyperbolic tangents (*tanh*) functions in Figure 7. These gates are used to decide which past information to be maintained and discarded when updating the current state. In the proposed method, we use a bi-directional LSTM, a construct of two LSTM. There are 1000 hidden states for each LSTM layer. 

#### 3.3.7. Loss Function 

The proposed architecture computes the loss by finding the conditional probability of the pose, the translation $\Gamma_{k}$ and rotation ${Ọ}_{k}$, given a consecutive LiDAR $L_{d}$ and camera $C_{a}$ scan at time t, as shown in (10) and (11):

(10) $$P\left({\;\Gamma }_{k}\;|\;\left(L_{d}\;\wedge \;C_{a}\right)\right)=p\left(\Gamma_{k_{1,\ldots }}\Gamma_{k_{t}}/\;(L_{d_{1,\ldots }}\;L_{d_{t}}\right)\;\wedge \;\left(C_{1,\ldots }\;C_{a_{t}}\right)),\;$$

(11) $$P\left(\;{Ọ}_{k}\;|\;\left(L_{d}\;\wedge \;C_{a}\right)\right)=p\left(\;{Ọ}_{k_{1,\ldots }}{Ọ}_{k_{t}}/\;(L_{d_{1,\ldots }}\;L_{d_{t}}\right)\;\wedge \;\left(C_{1,\ldots }\;C_{a_{t}}\right)),$$

The optimal parameter (θ) of the network is derived from:

(12) θΓ= (  θ argmax p ( Γk )t | (Ld ∧ Ca)t ) ,

(13) θỌ=(  θ argmax p ( Ọk )t | (Ld ∧ Ca)t ) ,

where $\theta^{\Gamma }$ and $\theta^{Ọ}$ are the optimal parameters for translation and orientation, respectively. 

The loss function is calculated using mean square error (MSE) by minimising the Euclidean distance between the ground truth pose (translation $\Gamma_{k}$ and rotation ${Ọ}_{k}$) and the predicted pose (${Ѓ}_{k\;}$, ${Ộ}_{k}$) at a given time t, as shown in (14):

(14) θ*= θ argmin 1N ∑i=1N|| Ѓk− Γk|| 22+ϰ || Ộk−Ọk|| 22, 

where ∥·∥ is 2-norm, ϰ is a scale factor to balance the weight of rotation and N is the number of samples. We use similar scale factor as Ref. [21].

## 4. Results

This section provides a detailed description of the proposed method of training and evaluation. As described in the methodology section, we implement three different models and evaluate their performance using the publicly available KITTI dataset and our LboroAV2 dataset collected for SLAM-related research. We compare our results against single-sensor-based and fusion of LiDAR and camera-based recent work.

### 4.1. Training 

The LboroAV2 dataset and the KITTI VO/SLAM benchmark [35] are used to evaluate the proposed method. The KITTI VO/SLAM benchmark contains 22 images and LiDAR data sequences. Only 11 of the KITTI sequences (00–10) have ground truth. The other sequences (11–21) only contain raw sensory data. This benchmark is very challenging for VO algorithms because of its urban scenery with maximum speeds of up to 90 km/h with numerous dynamic objects within each frame, all while being recorded at a relatively low frame rate (10 fps).

Because only the ground truths for sequences 00–10 are provided in the KITTI benchmark, the relatively long sequences 00, 01, 02, 06, 07, 08 and 10 from KITTI and sequences 03 and 04 from LboroAV2 are used for training, and the KITTI sequences 03, 04, 05, 09 and LboroAV2 sequences 01 and 02 are used for testing. To generate more samples for both raw images and LiDAR data, each trajectory is segmented into a random sequence length between the range of [5,7] for training and length of 6 for testing. However, due to this randomness, duplicated segments are inevitable and are, therefore, removed if a generated segment is already presented within the augmented dataset. This transformation is applied on both datasets used.

One of the major challenges in using deep learning architecture for any application is optimization of the mode that determines the effectiveness and generalization ability of the algorithm. There are two major factors that effectively improve generalization of deep learning algorithms. The first, regularization, is the effective way to improve the generalization capacity of a deep learning model [43,44], and the second is use of diverse data that effectively represent the real environment [45]. We use Adagrad optimizer, which determines the best parameter to minimize the loss function. As the data come from two streams, the Adagrad optimizer provides a boost to the SGD ability to adapt values in a manner conforming with the history of gradients before and after the fusion. We also use the LboroAV2 dataset to increase the generalization of the model. As stated in dataset Section 3.1, the LboroAV2 data contain different environmental conditions and settings that are not included in the KITTI dataset. The studies take into account relevant hyperparameters, such as learning rate, optimization function, number of epoch batch size and sequence length, based on previous similar works. The final hyperparameters are chosen following considerable research using a variety of alternatives.

The network was implemented in PyTorch and trained using an NVIDIA GeForce 2080 T GPU. The network was trained for 50 epochs with a learning rate of 0.0005. The encoder designed is inspired by the FlowNet model [17]. However, in contrast to using a pre-trained model, the model parameters in our experiment were trained from scratch. To prevent the model from overfitting, the batch normalization and dropout layers are employed at every 2D convolutional layer. To reduce the size of the high-dimensional point-cloud data, average pooling is employed at every 1D convolutional layer.

We experimented our approach in three different methods:

- RGB camera image (denoted as Proposed Camera)
- Fusion of RGB camera image and LiDAR point cloud data (denoted as Proposed_FCL_pcd)
- Fusion of RGB camera image and projection of RGB LiDAR panoramic projection image (denoted as Proposed_FCL_2d).

Table 1 presents the root mean square error (RMSE) of the translation and rotation for all subsequent lengths between 100 and 800 m with a varying speed among each sequence. The proposed method’s quantitative result is based on the KITTI SLAM/VO evaluation procedure. 

The training and testing dataset contains a mixture of moving and static objects captured at 10 fps with the car moving at different speeds. Our average rotation and translation error on the testing sequences while using the KITTI dataset shows that all three proposed methods outperform Deep VO and Viso2_m algorithms.

**Figure 8:**
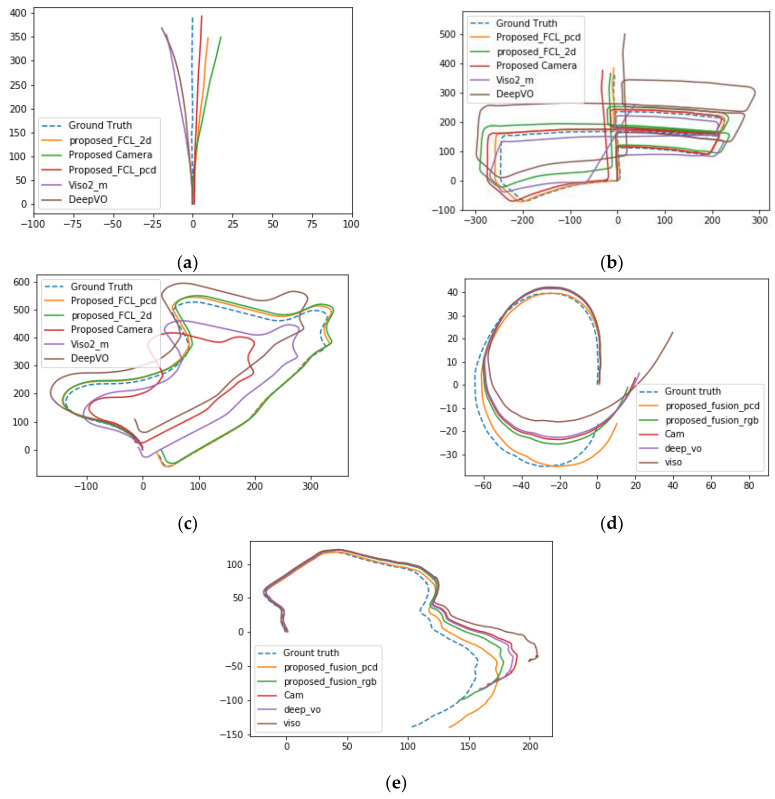
Results of the proposed algorithm’s trajectory (measured in meters) on the testing sequence. (**a**–**c**) Results with KITTI dataset sequence 04, 05 and 09, respectively; (**d**,**e**) results with sequence 01 and 02 of LboroAV2 dataset.

The result shows that the rotation and translation in the LboroAV2 dataset is higher than that of KITTI. This is because the LboroAV2 dataset is collected at a lower speed, less than 5 km/h. It is also evident in the results, as presented in Figure 8, that the fusion of LiDAR and camera outperforms the single modality techniques in all sequences. This proves that the proposed network can effectively learn the ultimate way to fuse the extracted RGB camera and LiDAR feature at the compressed representation node. The result also confirms that the fused features contain relevant temporal information, which helps the RNN network to learn the progressive relationship between consecutive scans and, hence, the errors caused by drift decrease.

**Figure 9:**
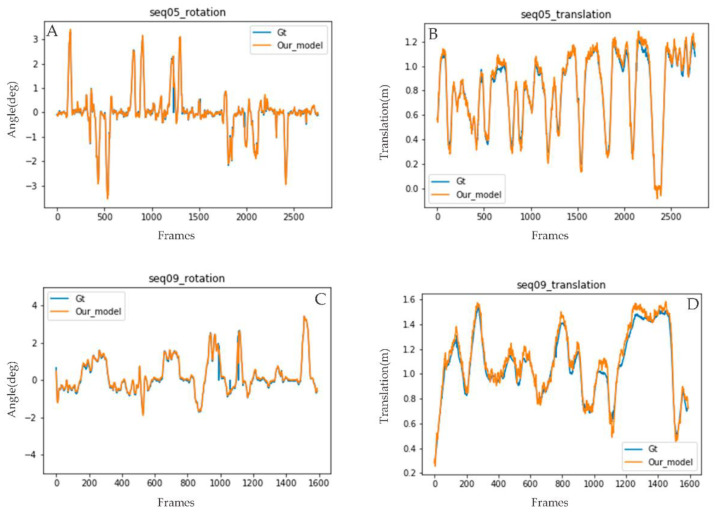
Results of our proposed method’s estimated translation and rotation plotted against the ground truth (results from KITTI 05 (**A**,**B**) and 09 (**C**,**D**) used as a sample).

**Figure 10:**
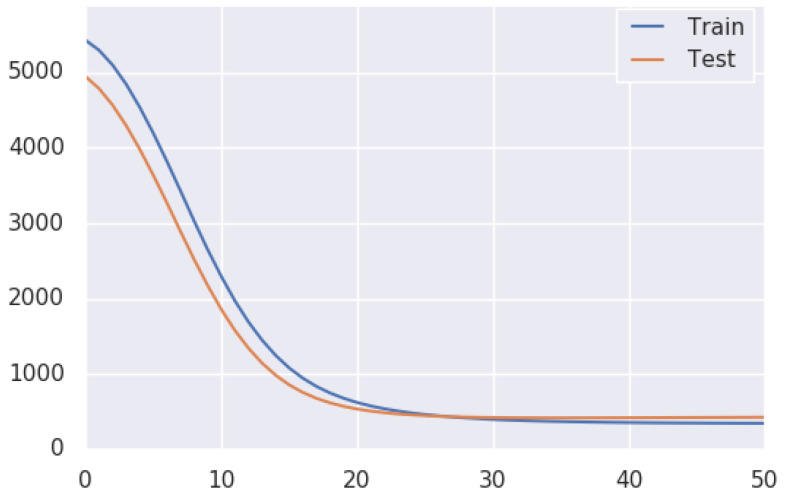
Training and test loss curve of the proposed method.

It is also evident in Figure 9 that estimated trajectory errors are not evenly distributed throughout each sequence. The result shows that most translation and rotation occurred around turns. When the car approaches turns, it reduces speed or comes to a stop. The amount of training data on KITTI while the car is driving lower than 20 km/h and greater than 50 km/h is minimal; errors are observed higher in this region. A major part of the translation error originates when the car is driven at a speed of more than 50 km/h or when the translation between two consultive frames is more than 1 m, as shown in Figure 9B,D. The proposed method loss function curve is presented in Figure 10.

### 4.2. Results Evaluations

Furthermore, a significant amount of KITTI and LboroAV2 datasets are collected while the car is driven on a straight road; hence, the number of corners and turns in training is limited. One of the contributions of LboroAV2 is its richness in the slow-moving training dataset. While this dataset helps to improve the model by providing a slow-moving dataset, LboroAV2 has few corners. An extensive training dataset that represents all driving scenarios in the environments is required to increase the robustness of deep-learning-based odometry algorithms. The images and LiDAR scans are captured at 10Hzin for both datasets. Hence, there is a higher motion displacement between consecutive frames, especially when the car travels at a higher speed. This is an additional challenge for the model as there will be no features to extract, especially driving in open areas, such as highways.

As presented in Table 2, the proposed method yields a training and testing time reduction compared to the DeepVo architecture. This is due to the proposed method’s robustness in extracting useful geometrical and temporal features to a size of 250-dimension compressed representation node. Hence, the trainable parameter passed to the RNN is smaller than similar methods.

### 4.3. Explainable Representation

Hence, our feature extractions are accompanied by a compressed representation node. One of the contributions of the proposed architecture is that it opens the door for odometer researchers to explore the compressed representation node. One of the major drawbacks of deep neural architecture is that the hidden neurons’ learning process is unknown [46,47,48]. Visualising the compressed node during training time provides access to gain insight into the neural network’s black box, which makes it possible to observe and analyse the learning process of the model.

**Figure 11:**
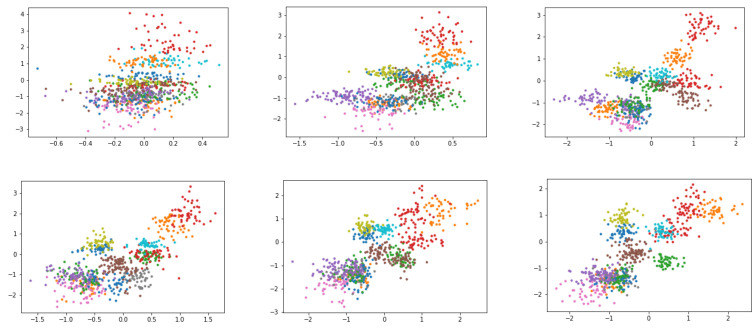
Visualisation of the compressed representation through the training cycle. Each colour represents elements in the compressed dimension. This result was obtained by training the model with a 16-dimensional compressed representation to make the visualisation more expressive.

Even though exploring the black box of deep learning architecture is not the focus of this paper, the authors are interested in expressing the proposed method’s contribution to attaining explainable AI. We re-train the model using a 16-dimensional compressed representation to make visualisation of the compressed space more expressive. Figure 11 displays the learning process of the proposed architecture through the training period. We took a sample of six images between the range of the first iteration to the last. The result shows that each of the 16 dimensions is learning different features. The model is learning how to differentiate feature spaces between each dimension whenever the learning time is increased.

This paper aims to highlight the proposed architecture’s potential to shed light on the hidden box of deep learning. Moreover, investigation of the compressed representation node will help to fine-tune the model by omitting redundant dimensions. Further research is also required to understand individual compressed representation nodes and their contribution towards increasing or decreasing translation rotation accuracy. A full investigation of the interpretation, explainability and usage of compressed dimensions is the future research direction of the authors.

## 5. Conclusions

This study presented deep-learning-based fusion of LiDAR and camera architecture for odometry application. Our model consists of an encoder designed to extract optical flow between two frames, a compressed representation node of the input and the RNN model. Besides effectively down-sampling the input features, the proposed approaches extracted useful geometrical information while also learning to extract suitable features for the RNN to learn temporal information. We also contributed a unique dataset that can be used for a range of SLAM and odometry research. We benchmarked the proposed method on the KITTI and LboroAV2 datasets, and our fusion approach significantly decreases translation and rotation error compared to both single modality and fusion-based algorithms. Our approach substantially decreases training time whilst using high-dimensional data from two different sources.

Besides better accuracy, the compressed representation node of the proposed method opens the door for future research to explore the black box of deep learning architecture. Further examination is also essential to understand specific compressed representation nodes and their role in improving translation rotation accuracy.

## Figures and Tables

**Table 1:** Comparison of RMSE between the proposed method and benchmarking approaches.

Dataset		KITTI	KITTI	KITTI	Mean KITTI	LboroAV2	LboroAV2	Mean_lboroAV2
Sequence		4	5	9		1	2	
DeepVO	$\Gamma_{k}$	7.19	2.62	8.11	5.97	9.56	9.86	9.71
	${Ọ}_{k}$	6.97	3.61	8.83	6.47	10.2	10.41	10.3
VISO2_M	$\Gamma_{k}$	4.69	19.22	41.56	21.82	44.63	38.21	41.42
	${Ọ}_{k}$	4.49	17.58	32.99	18.35	35.8	42.11	38.95
SFMLearner monocular supervised	$\Gamma_{k}$	10.86	16.76	22.27	16.63	NA	NA	NA
	${Ọ}_{k}$	5.13	4.06	3.62	4.27	NA	NA	NA
UnMono Monocular	$\Gamma_{k}$	2.15	3.84	5.59	3.86	NA	NA	NA
	${Ọ}_{k}$	0.89	1.29	2.57	1.58	NA	NA	NA
Our camera only	$\Gamma_{k}$	2.33	2.45	9.34	4.7	8.24	10.53	9.38
	${Ọ}_{k}$	3.25	2.56	10.7	5.5	9.11	10.61	9.86
Our fusion using LiDAR 2D image and camera	$\Gamma_{k}$	2.23	1.89	1.77	1.96	7.94	3.69	5.81
	${Ọ}_{k}$	2.33	1.9	1.83	2.02	7.29	3.27	5.28
proposed (Fusion using LiDAR point cloud and camera)	$\Gamma_{k}$	2.01	1.75	1.7	1.82	3.3	3.43	3.36
	${Ọ}_{k}$	2.11	1.45	1.78	1.178	3.14	3.62	3.38

$\Gamma_{k}$ Average translation drift RMSE (%) on length of 100–800 m; ${Ọ}_{k}$ average rotation RMSE drift (^0^/m) on length of 100–800 m. The SFMLearner and UnMono translation and rotation errors on KITTI are directly used from the papers.

**Table 2:** Comparison of the proposed method and Deep_VO training and testing time on the KITTI dataset.

Method	Avg. Training Time/Epoch (Minutes)	Avg. Testing Time/Epoch (Minutes)
Deep_VO *	4.25	0.52
Proposed_camera	1.51	0.28
proposed FCL_2d	2.18	0.34
proposed FCL_pcd	2.25	0.35

* Since no official DeepVO publicly available code for benchmarking exists, we test our result by implementing the architecture as proposed in Ref. [21].

## References

[1] Tibebu H., Roche J., De Silva V., Kondoz A. (2021). LiDAR-based glass detection for improved occupancy grid mapping. Sensors.

[2] Zhai G., Liu L., Zhang L., Liu Y., Jiang Y. (2020). PoseConvGRU: A Monocular Approach for Visual Ego-motion Estimation by Learning. Pattern Recognit..

[3] Briod A., Zufferey J.C., Floreano D. (2016). A method for ego-motion estimation in micro-hovering platforms flying in very cluttered environments. Auton. Robots.

[4] Vicente P., Jamone L., Bernardino A. (2016). Robotic Hand Pose Estimation Based on Stereo Vision and GPU-enabled Internal Graphical Simulation. J. Intell. Robot. Syst. Theory Appl..

[5] Liu J., Ding H., Shahroudy A., Duan L.Y., Jiang X., Wang G., Kot A.C. (2020). Feature Boosting Network for 3D Pose Estimation. IEEE Trans. Pattern Anal. Mach. Intell..

[6] Pandey T., Pena D., Byrne J., Moloney D. (2021). Leveraging deep learning for visual odometry using optical flow. Sensors.

[7] Teixeira B., Silva H., Matos A., Silva E. (2020). Deep Learning for Underwater Visual Odometry Estimation. IEEE Access.

[8] Liu Q., Zhang H., Xu Y., Wang L. (2020). Unsupervised deep learning-based RGB-D visual odometry. Appl. Sci..

[9] Liu Q., Li R., Hu H., Gu D. (2019). Using Unsupervised Deep Learning Technique for Monocular Visual Odometry. IEEE Access.

[10] Li B., Hu M., Wang S., Wang L., Gong X. Self-supervised Visual-LiDAR Odometry with Flip Consistency. Proceedings of the IEEE/CVF Winter Conference on Applications of Computer Vision (WACV) 2021.

[11] Klein G., Murray D. Parallel tracking; mapping for small AR workspaces. Proceedings of the 2007 6th IEEE and ACM International Symposium on Mixed and Augmented Reality ISMAR.

[12] Mur-Artal R., Tardos J.D. (2017). ORB-SLAM2: An Open-Source SLAM System for Monocular, Stereo, and RGB-D Cameras. IEEE Trans. Robot..

[13] Newcombe R.A., Lovegrove S.J., Davison A.J. DTAM: Dense tracking and mapping in real-time. Proceedings of the IEEE International Conference on Computer Vision.

[14] Engel J., Schöps T., Cremers D. (2014). LSD-SLAM: Large-Scale Direct monocular SLAM. Computer Vision—ECCV 2014.

[15] Duan C., Junginger S., Huang J., Jin K., Thurow K. (2019). Deep Learning for Visual SLAM in Transportation Robotics: A review. Transp. Saf. Environ..

[16] Yang X., Li X., Guan Y., Song J., Wang R. (2020). Overfitting reduction of pose estimation for deep learning visual odometry. China Commun..

[17] Dosovitskiy A., Fischer P., Ilg E., Hausser P., Hazirbas C., Golkov V., van der Smagt P., Cremers D., Brox T. FlowNet: Learning optical flow with convolutional networks. Proceedings of the IEEE International Conference on Computer Vision.

[18] Parisotto E., Chaplot D.S., Zhang J., Salakhutdinov R. Global pose estimation with an attention-based recurrent network. Proceedings of the IEEE Computer Society Conference on Computer Vision and Pattern Recognition Workshops.

[19] Muller P., Savakis A. Flowdometry: An optical flow and deep learning based approach to visual odometry. Proceedings of the 2017 IEEE Winter Conference on Applications of Computer Vision, WACV 2017.

[20] Costante G., Mancini M., Valigi P., Ciarfuglia T.A. (2016). Exploring Representation Learning With CNNs for Frame-to-Frame Ego-Motion Estimation. IEEE Robot. Autom. Lett..

[21] Wang S., Clark R., Wen H., Trigoni N. DeepVO: Towards end-to-end visual odometry with deep Recurrent Convolutional Neural Networks. Proceedings of the IEEE International Conference on Robotics and Automation.

[22] Costante G., Ciarfuglia T.A. (2018). LS-VO: Learning Dense Optical Subspace for Robust Visual Odometry Estimation. IEEE Robot. Autom. Lett..

[23] Zhang J., Singh S. (2015). LOAM: LiDAR Odometry and Mapping in Real-time. Robot. Sci. Syst..

[24] Shan T., Englot B. LeGO-LOAM: Lightweight and Ground-Optimized LiDAR Odometry and Mapping on Variable Terrain. Proceedings of the 2018 IEEE/RSJ International Conference on Intelligent Robots and Systems (IROS).

[25] Velas M., Spanel M., Herout A. Collar Line Segments for fast odometry estimation from Velodyne point clouds. Proceedings of the IEEE International Conference on Robotics and Automation.

[26] Velas M., Spanel M., Hradis M., Herout A. CNN for IMU assisted odometry estimation using velodyne LiDAR. Proceedings of the 18th IEEE International Conference on Autonomous Robot Systems and Competitions, ICARSC 2018.

[27] Li Q., Chen S., Wang C., Li X., Wen C., Cheng M., Li J. Lo-net: Deep real-time LiDAR odometry. Proceedings of the IEEE Computer Society Conference on Computer Vision and Pattern Recognition.

[28] Lu W., Zhou Y., Wan G., Hou S., Song S. L3-net: Towards learning based LiDAR localisation for autonomous driving. Proceedings of the IEEE Computer Society Conference on Computer Vision and Pattern Recognition.

[29] Cho Y., Kim G., Kim A. Unsupervised Geometry-Aware Deep LiDAR Odometry. In Proceedings of IEEE International Conference on Robotics and Automation.

[30] Lu W., Wan G., Zhou Y., Fu X., Yuan P., Song S. DeepVCP: An end-to-end deep neural network for point cloud registration. Proceedings of the IEEE International Conference on Computer Vision.

[31] Zhang J., Singh S. Visual-LiDAR odometry and mapping: Low-drift, robust, and fast. Proceedings of the IEEE International Conference on Robotics and Automation.

[32] Graeter J., Wilczynski A., Lauer M. LIMO: LiDAR-Monocular Visual Odometry. Proceedings of the IEEE International Conference on Intelligent Robots and Systems.

[33] Yue J., Wen W., Han J., Hsu L.-T. (2020). LiDAR Data Enrichment Using Deep Learning Based on High-Resolution Image: An Approach to Achieve High-Performance LiDAR SLAM Using Low-cost LiDAR. arXiv.

[34] Guizilini V., Li J., Ambrus R., Pillai S., Gaidon A. (2019). Robust Semi-Supervised Monocular Depth Estimation with Reprojected Distances. PMLR Proc. Mach. Learn. Res..

[35] Geiger A., Lenz P., Stiller C., Urtasun R. (2013). Vision meets robotics: The KITTI dataset. Int. J. Rob. Res..

[36] Roche J., De-Silva V., Kondoz A. (2021). A Multimodal Perception-Driven Self Evolving Autonomous Ground Vehicle. IEEE Trans. Cybern..

[37] Valente M., Joly C., De La Fortelle A. (2019). An LSTM network for real-time odometry estimation. IEEE Intell. Veh. Symp. Proc..

[38] Restrepo R. LiDAR Data to 2D. http://ronny.rest/blog/post_2017_03_25_LiDAR_to_2d/.

[39] Krizhevsky A., Sutskever I., Hinton G.E. (2017). ImageNet classification with deep convolutional neural networks. Commun. ACM.

[40] Simonyan K., Zisserman A. Very deep convolutional networks for large-scale image recognition. Proceedings of the 3rd International Conference on Learning Representations, ICLR 2015-Conference Track Proceedings.

[41] Valente M., Joly C., La Fortelle A.D. Deep Sensor Fusion for Real-Time Odometry Estimation. Proceedings of the IEEE International Conference on Intelligent Robots and Systems, The Venetian Macao.

[42] Greff K., Srivastava R.K., Koutnik J., Steunebrink B.R., Schmidhuber J. (2017). LSTM: A Search Space Odyssey. IEEE Trans. Neural Networks Learn. Syst..

[43] Zheng Q., Yang M., Yang J., Zhang Q., Zhang X. (2018). Improvement of Generalization Ability of Deep CNN via Implicit Regularization in Two-Stage Training Process. IEEE Access.

[44] Zhang S., Qian Z., Huang K., Wang Q., Zhang R., Yi X. (2021). Towards Better Robust Generalization with Shift Consistency Regularization. PMLR Proc. Mach. Learn. Res..

[45] Ranftl R., Lasinger K., Hafner D., Schindler K., Koltun V. (2022). Towards Robust Monocular Depth Estimation: Mixing Datasets for Zero-Shot Cross-Dataset Transfer. IEEE Trans. Pattern Anal. Mach. Intell..

[46] Buhrmester V., Münch D., Arens M. (2021). Analysis of Explainers of Black Box Deep Neural Networks for Computer Vision: A Survey. Mach. Learn. Knowl. Extr..

[47] Shao Y., Cheng Y., Shah R.U., Weir C.R., Bray B.E., Zeng-Treitler Q. (2021). Shedding Light on the Black Box: Explaining Deep Neural Network Prediction of Clinical Outcomes. J. Med. Syst..

[48] Liang Y., Li S., Yan C., Li M., Jiang C. (2021). Explaining the black-box model: A survey of local interpretation methods for deep neural networks. Neurocomputing.

